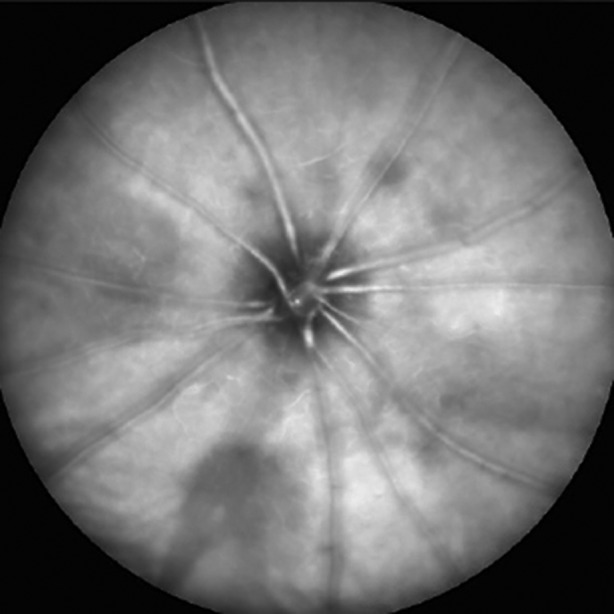# Microglia alterations predict neurodegeneration in glaucoma

**Published:** 2015-05-01

Glaucoma is a progressive neurodegenerative disorder that affects the retinal ganglion cells and their axons. Its symptoms become manifested only at late disease stages, when vison loss is irreversible. Thus, identifying early biomarkers of degeneration is central to make therapeutic interventions possible. Alterations of microglia are detectable early during glaucoma progression; however, whether these early changes can predict the severity of late neurodegeneration in the retina is unknown. To address this, Alejandra Bosco and colleagues used a mouse model of inherited glaucoma in which microglial cells were genetically labelled with green fluorescent protein (GFP) expressed under the control of the fractalkine receptor locus (*Cx3cr1*). By using confocal ophthalmoscopy, the authors imaged microglia in young mice and correlated these data with the severity of optic nerve pathology in the same animals 5 to 10 months later. Interestingly, the authors found that early microglial activation and microgliosis significantly correlate with late retinal neurodegeneration. These changes might thus predict disease severity and could be visually monitored to guide therapeutic interventions. **Page 443**

**Figure:**